# The hospital sink drain microbiome as a melting pot for AMR transmission to nosocomial pathogens

**DOI:** 10.1038/s44259-025-00137-9

**Published:** 2025-07-29

**Authors:** Gregory E. McCallum, James P. J. Hall

**Affiliations:** Department of Evolution, Ecology and Behaviour, Institute of Infection, Veterinary and Ecological Sciences, University of Liverpool, Liverpool, UK

**Keywords:** Microbial ecology, Antimicrobials, Environmental microbiology, Pathogens

## Abstract

The hospital sink drain microbiome can harbour opportunistic pathogens and antimicrobial resistance genes (ARGs). Aspects of this habitat, such as exposure to disinfectants, antibiotics, nutrients, and body fluids could exacerbate horizontal gene transfer of ARGs and clinically impactful pathogen resistance. Here, we explore features of the hospital sink drain that may favour ARG acquisition and transmission, highlight studies providing evidence of transfer, and consider strategies to mitigate these risks.

## Introduction: the evolution of resistant opportunistic pathogens

One of the greatest concerns in the rise of antimicrobial resistance (AMR) is the increase of multidrug resistance in opportunistic pathogens: species that are usually non-pathogenic when encountering healthy individuals but can cause severe infections in vulnerable populations. The risks posed by multidrug-resistant opportunistic pathogens is evident in the 2024 World Health Organisation Bacterial Priority Pathogens List, where, of the 15 species or groups named, 10 are generally considered opportunistic pathogens. These include drug-resistant strains of *Acinetobacter baumannii* and Enterobacterales in the critical group, as well as *Enterococcus faecium*, *Pseudomonas aeruginosa*, and *Staphylococcus aureus* in the high-priority group^1^. Because opportunistic pathogens exist in large populations outside of an infection context, such as in the environment or as human commensals, evolutionary processes occurring in these natural habitats shape the risks subsequently posed by opportunistic pathogens when they do cause disease. In a hospital setting, where the direct threat of opportunistic infection is already elevated for patients who may be immunocompromised or have other underlying health conditions, microbial evolution in response to complex environmental selection pressures can facilitate the emergence of difficult-to-treat pathogens^2^.

Antimicrobial resistance can arise in pathogens through de novo mutations, and through horizontal gene transfer (HGT) of antimicrobial resistance genes (ARGs), which is often mediated by mobile genetic elements (MGEs)^3^. This ability to acquire resistance is crucial for understanding the rapid rise of AMR among opportunistic pathogens, because many of the clinically important ARGs are often found on MGEs. For example, plasmid-disseminated *mcr* genes confer resistance to colistin^4^, while carbapenem resistance genes are often carried on plasmids which have been responsible for the rapid global dissemination of carbapenemases, and the rise of infections caused by carbapenemase-producing Enterobacterales (CPEs)^5^. Importantly, MGEs can often provide resistance to multiple antimicrobials at once because multiple ARGs can be present on a single MGE and transfer together in a single event. Subsequent selection for just one of these traits can result in other resistance traits rising to high frequency without direct selection, thanks to genetic linkage^6,7^. In other cases, resistances can spread across a population solely through the ability of the MGE to act as an infectious element^8^. Owing to the activity of MGEs, opportunistic pathogens might gain resistance to multiple antimicrobials in their natural habitats without ever experiencing antibiotic treatment in an infection context. Understanding the ecology of opportunistic pathogens and their cognate MGEs—and identifying how and where organisms acquire clinically-relevant resistance traits—could focus and inform strategies to impede HGT in these habitats to reduce the threat of subsequent difficult-to-treat infections.

## The microbiome of the built environment

Microbiomes are microbial ecosystems, with the term ‘microbiota’ describing the microbes present, and the term ‘microbiome’ describing the microbes, DNA, and environmental conditions^9^. While the human gut microbiome has been widely explored as an extensive reservoir for ARGs and opportunistic pathogens—and is undoubtedly an important location for HGT events^10^—environmental microbiomes, including the microbiome of the built environment, may also play a significant role in the spread of ARGs and emergence of resistant pathogens. The ‘built environment’ describes human-built areas including buildings and any other manmade structures or systems. The relative inhospitality of these environments to microbes, due to the lack of water and nutrients, means that the microbiomes found in the built environment are distinct to those found in nature. The unique habitats formed by the built environment, both physically and chemically, cause the resident microbes to face selective pressures that few species can survive and thrive in, making them less diverse than some other environmental microbiomes (e.g. those of soil) as a consequence^2,11^.

The development of next-generation sequencing technologies in recent decades has rapidly advanced the study of microbiomes. Metagenomic sequencing, where all DNA in a sample is extracted and sequenced^12^, has allowed a comprehensive view of the taxonomic profiles of a sample without the need for culturing, as well as revealing the genes and MGEs present^13^. Microbiome studies employing these techniques, and others, have indicated that humans are the primary source of microbes in the built environment^14^. However, the microbial community is influenced by the types of activity occurring. For example, in domestic settings, the presence of pet cats or dogs increases bacterial richness and diversity in a household^15^, and studies sampling kitchens often find foodborne pathogens like *Campylobacter*, especially when unhygienic handling of food has occurred^16,17^. In the hospital built environment, human presence has a profound impact on microbiome composition. A study tracking the microbiomes of surfaces in a hospital found a distinct shift in community composition once the hospital became operational, with the bacterial communities of room surfaces converging to increasingly resemble those of their resident patients over time^18^. Similarly, the microbiomes of bedrails, computer keyboards, and sinks in an intensive care unit (ICU) shifted to more environmental-associated bacteria such as Bacillaceae and Rhizobiaceae during renovations, and back to more human-associated microbes like *Staphylococcus* and *Cutibacterium* after re-opening^19^.

As well as microbes, the built environment can act as a reservoir of ARGs^13^. The contribution of hospital ARGs to global environmental ARG load is likely to be relatively small compared with contributions from agricultural and community inputs^20,21^. However, the presence of patients who are likely to have compromised immunity heightens the risks posed by the microbiome of the hospital built environment, which can act as a source of both ARGs and opportunistic pathogens that can lead to hospital-acquired infections^22^. Within days of occupying a room, bacteria and ARGs originating from a patient can disseminate into the hospital environment^23^. Likewise, patients can be colonised by microbes originating from the hospital environment. For example, there is overlap between the preterm infant gut microbiome and the hospital room microbiome, where exchange of strains between the infant and room have been documented in both directions^24^. Within the hospital built environment there are numerous microbiomes that patients and staff may encounter, such as those present on surfaces like the floor and door handles^18,25^. Of these, the concealed microbiomes of sink and shower drains contain the greatest non-human-associated microbial biomass in the hospital built environment^23,26,27^. This ‘grey water’ microbiome located immediately downstream of water outlets therefore has the potential to be an important reservoir for the evolution and dissemination of clinically relevant opportunistic pathogens.

## Features of the sink drain microbiome promoting resistance gene exchange

**Fig. 1:**
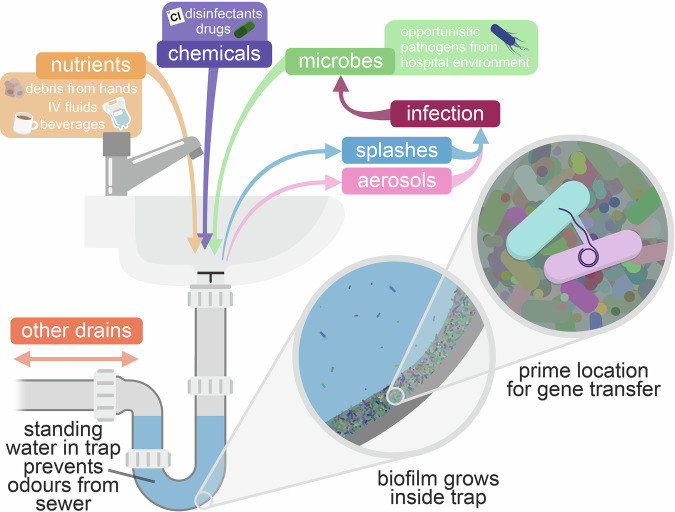
The hospital sink drain microbiome as a melting pot for AMR transmission to nosocomial pathogens.

Sink traps are plumbing devices designed to prevent foul odours from emanating from the sewer by placing a pool of standing water immediately downstream of the waste outlet (Fig. 1). Water exiting the sink flushes and re-fills the trap. Sink traps therefore offer a moist and aerated surface in which a microbiome can develop. Whilst all microbiomes in the hospital setting provide potential for the spread of opportunistic pathogens and ARGs, the hospital sink drain microbiome has features that may especially favour acquisition and transmission of ARGs. Firstly, sink traps are significantly more challenging to clean compared to other hospital surfaces, since they are located down a narrow and often curved pipe, usually partially obscured by a strainer installed to prevent larger objects from passing through and blocking the drain further downstream, which prevents physical disruption. Microbes attach and grow inside the drain as biofilms: surface-attached communities which are highly resistant to typical disinfection procedures^28^ and are considered hotspots for the maintenance, evolution, and transmission of resistance-encoding plasmids^29^. In a 2020 study by Ledwoch et al., the authors developed an in vitro sink drain biofilm model, and found that widely-used chorine-based disinfectants such as sodium hypochlorite (bleach) only partially reduced viability of bacteria in the model, and biofilms were able to recover just 4 days after disinfection^30^. Similarly, a study on real-world hospital sink drains found that biofilms could fully recover within 7 days of treatment with hydrogen peroxide or bleach^31^.

As well as failing to completely inhibit biofilms, disinfectants might actually stimulate emergence of AMR. Chlorine-based disinfectants were shown to promote ARG transmission by HGT both within strains of *Escherichia coli* and between *E. coli* and *Salmonella* when present at subinhibitory concentrations^32^. Other disinfectants, such as quaternary ammonium compounds (QACs)^33^ and triclosan^34^, have been shown to have similar effects, though it should be noted that triclosan is no longer used in hospital detergents in the European Union since 2017^35^. HGT increases after disinfectant exposure due to the production of reactive oxygen species, which triggers the SOS response—promoting both expression of ARGs and mobile genetic elements—and increases cell membrane permeability^32,36^. Disinfectants can also select for chromosomal mutations that provide direct cross-resistance to antimicrobials^37^, or promote AMR emergence indirectly by increasing conjugation permissiveness^34^. While harsh disinfectant stresses might transiently reduce the microbiological load, the survivors may pose an even greater threat.

Besides disinfectants, hospital sink traps can be exposed to diverse types of waste entering the sink, such as debris, oils, and dead cells from hands during washing, left over beverages, IV fluids, and drugs. These can act as sources of nutrients to facilitate bacterial growth, as triggers for HGT, and can impose selective pressures favouring resistance. When assessing different water chemistry dipsticks, Rodger et al. detected both tetracycline and sulphonamide antibiotics in a hospital sink trap^38^. The same group surveyed 287 sinks across 29 hospitals in the United Kingdom and detected antibiotic residues in 33% of sink trap samples, with the majority of detections being for beta-lactam antibiotics^39^. Another study detected antimicrobial residues in 31/115 hospital sink samples taken over a 12-week period^40^. Like disinfectants, exposure to low concentrations of some antibiotics is known to promote HGT^41^. Although most hospitals have strict rules on sink usage, including patient-facing sinks being designated to handwashing only, a study monitoring activity at four ICU sinks in the United States found that only 4% of total interactions with the sinks were for handwashing^42^. On top of the risk of increasing selective pressures in the sink drain, the use of patient-facing sinks for activity outside of handwashing has a risk of enriching the sink drain microbiome. Addition of nutrients into sink traps is crucial for the growth of some opportunistic pathogens, particularly CPEs^43^.

## The sink drain microbiome as a persistent reservoir of opportunistic pathogens and ARGs

Many studies have demonstrated that the hospital sink drain is a key, long-term, persistent reservoir for opportunistic pathogens and ARGs, and have suggested that it can act as a source of infection^44–63^. A systematic review by Kizny Gordon et al. in 2017 identified 32 studies reporting carbapenem-resistant bacteria in hospital water, with drains representing the most common location, and with *Pseudomonas aeruginosa* being the most frequent species^44^. Another systematic review by Volling et al. in 2020 identified 51 studies suggesting that sink drainage systems are a reservoir for hospital-acquired Gammaproteobacterial patient colonisation or infection, again implicating *P. aeruginosa* as the most common organism involved^45^. More recent reports describe similar patterns. Sink drains were proposed to be a major reservoir when investigating the sources of *Serratia marcescens* colonisation in a neonatal ICU, with an average of 44% of sink samples being positive for *S. marcescens* across five periods of sampling^55^. In a study that collected sink samples over 27 months in two hospitals and sequenced over 800 isolates, species of *Pseudomonas* and *Serratia* persisted for the whole sampling duration, as well as several ARG-carrying plasmids^56^. Genes encoding extended spectrum beta-lactamases (ESBLs), conferring resistance to beta-lactam antibiotics, are commonly found in the hospital sink drain. In a culture-based study sampling hospital surfaces in non-outbreak settings, 26.7% of sink drains contained ESBL-producing bacteria—a higher proportion than any other hospital surface^57^. During a long-term outbreak of carbapenemase-producing organisms on an ICU in a Belgian hospital, sink drains were a persistent reservoir, even withstanding disinfection attempts and replacement of contaminated equipment^58^. Using whole-genome sequencing, the authors found that the sink isolates carried the same multidrug resistance plasmid as the patient isolates, suggesting an epidemiological link. Interestingly, the plasmids also contained genes conferring resistance to QACs, which could explain why these strains could withstand disinfection attempts with QAC-based disinfectants^58^. Sinks were found to be a prolonged and recalcitrant reservoir of *Klebsiella pneumoniae* carbapenemase (KPC)-producing *E. coli* at a hospital in the United Kingdom^59^. The outbreak clone of carbapenem-resistant *E. coli* was identified in patient colonisation and infection, as well as in the sink drains. Multiple ward closures and replacement of sink traps and other plumbing infrastructure only resulted in temporary decline in incidence, as the sinks were rapidly recolonised^59^. In another case, sampling of sinks over a year in five different wards of a hospital found opportunistic pathogens to be highly prevalent, with *Pseudomonas* spp. and *Stenotrophomonas* being the most predominant, including multidrug-resistant *Pseudomonas aeruginosa*^60^. A further study describing an outbreak of extensively drug-resistant *P. aeruginosa* found the resistant strains persisting in the hospital environment, primarily in sinks and toilets, despite regular disinfection^61^. Even when a resistance gene has very low prevalence in the patient population, it can persist in the sink drain and potentially pose a risk to future patients. For example, an *Acinetobacter pittii* strain isolated from a sink drain in a Japanese hospital was found to harbour a plasmid carrying the ESBL gene *bla*_NDM-1_, despite no other New Delhi metallo-beta-lactamase (NDM)-producers being detected in any previous environmental or patient samples at the hospital^62^. In a study comparing sink drain biofilms in a neonatal ICU and an adult ICU in a French hospital, the authors found that the two wards had distinct bacterial communities: *Pseudomonas*, *Stenotrophomonas*, and *Staphylococcus* dominating the adult ICU; and *Achromobacter*, *Serratia*, and *Acidovorax* dominating the neonatal ICU. These differences were likely due to the regular disinfection regime at the neonatal ICU. Despite the differences in the communities, the ARG profiles of the sink communities on both wards showed no significant differences, indicating that the ARG abundance is independent of the taxonomic identities of the microbiota^63^. Whilst most studies have focused on the bacterial portion of the sink drain microbiota, it should be noted that hospital sink drains also act as a reservoir for eukaryotic opportunistic pathogens^64^ (though the risk of trans-species ARG HGT is likely smaller for eukaryotes).

In some cases, the causal role of the sink reservoir in driving an outbreak has been inferred by the success of interventions targeting sinks or sink use. A year-long outbreak of *bla*_OXA-48_-positive *Serratia marcescens* at an ICU in 2016 was not contained until the sink traps were identified as the source^65^. With a total of 34 cases and 3 patient deaths attributed to infection by the outbreak *S. marcescens*, all environmental samples were negative for the outbreak strains except for the sink outlets and traps. It was found that tap water was sometimes being used to clean the patients or flush gastric tubes, which the authors postulated led to contamination from the sinks and transmission of *S. marcescens*. Efforts to both replace and disinfect the traps failed to stop the outbreak, but behavioural changes after workshops for the staff to raise awareness of the risks of hospital sinks were successful in outbreak containment^65^. An outbreak of ESBL-producing *Klebsiella oxytoca* at a Canadian ICU was attributed to the handwashing sink drains after isolates, deemed identical by pulsed-field gel electrophoresis (PFGE), were found in patient and environmental samples. No new infections followed after replacement of all sink traps and increased sink disinfection regimes^66^. Another outbreak of NDM-producing *K. pneumoniae* on a surgical ICU in France was associated with the sinks, as the epidemic strain, consistent with 9 patient isolates based on PFGE, was isolated from a sink. An NDM-producing *Enterobacter cloacae* was also found in another sink. The outbreak was only halted after installation of new sink traps designed to reduce production of aerosols^67^. During a 2-year outbreak of *K. oxytoca* carrying *bla*_IMP-8_ at an ICU in Spain, a strain consistent with the outbreak strain via PFGE was detected in the drainpipe and trap of one sink located in a storage room. All other environmental and staff screening samples were negative. Permanent removal of the *K. oxytoca*-positive sink resulted in reduced cases, but the outbreak continued with new cases clustering in a cubicle adjacent to the storage room. The outbreak was finally eradicated after removal of the horizontal drainage system that had previously connected the sink in the cubicle to the removed sink in the storage room, indicating that connection between waste pipes could allow transmission of bacteria between sinks^68^. A narrative 5-year review of 19 studies reporting drain-associated outbreaks and interventions indicated that control measures were ultimately successful at stopping the outbreak in all cases where results were reported^69^.

Many of the studies described above provide circumstantial evidence linking outbreaks to the sink drain microbiome, and their observational design can struggle with proving a causal, directional link between an environmental reservoir and patient infection. Though an increasing number provide genomic data necessary for high-resolution identification of outbreak clones in the environment, most start after an outbreak has begun, introducing the possibility that the environmental reservoir has been contaminated by material from an infected patient, or from a shared third party, rather than acting as a source for infection. However, laboratory studies show that microbes inside sink traps *can* be readily transmitted out to the surrounding environment. Kotay et al. added green fluorescent protein (GFP)-expressing *E. coli* to a sink trap. Although running water into the sink did not disperse the bacteria directly from the trap, the authors showed that when a small amount of nutrients was added, the *E. coli* biofilm could grow upwards in the tailpipe at 1 inch per day, and reach the strainer in a week, causing dispersal when operating the faucet^70^. Such a design mimics a real hospital environment, where the biofilm inside a sink trap can mature for long periods of time with small amounts of nutrients entering regularly. Another study showed that droplets containing carbapenem-resistant Enterobacteriaceae could travel up to 1 metre from the sink to surrounding areas, and that sink and drain design could affect this droplet dispersal^71^. Improved drainage rates and avoidance of placing the drain directly under the tap both resulted in reduced dispersion, although it still could not be fully prevented^71^. These and other studies provide compelling evidence that bacteria in the sink trap microbiome can pose a threat, with opportunistic pathogens able to be dispersed to the surrounding environment, potentially risking patient colonisation and infection.

## The sink drain microbiome as a melting pot for resistance gene transmission

HGT of mobile genetic elements in a hospital setting allows clinically important ARGs to rapidly disseminate between opportunistic pathogens, and between persistent environmental microbes and opportunistic pathogens. A landmark study focussing on KPC-positive Enterobacteriaceae isolates in a hospital over 5 years found that *bla*_KPC_ was being carried by 62 different strains of 13 different species, which included 8 different genera^72^. The authors showed that *bla*_KPC_ spread in the hospital at multiple genetic levels, from strains transferring between patients, to transfer of plasmids between species, to transposition of ARG-harbouring transposons between plasmids. Clearly, HGT was important for the transmission of this ARG^72^, and the results hinted at a large, unsampled network of genetic exchange occurring outside of an infection context. Subsequent work sampling six sinks in the same hospital identified 14 distinct taxonomic clades with *bla*_KPC_ resident in the sink drain microbiome, with genomic analyses implicating sink-specific plasmid conjugation and transposition of *bla*_KPC_ from plasmids to chromosomes^73^. In a 2018 study, Weingarten et al. collected and sequenced isolates from patients and environmental locations over a 2 year period, and showed that hospital sinks were a vast reservoir for opportunistic pathogens^74^. The authors found isolates of *K. oxytoca* in the ICU sink that were identical to a patient isolate collected a year earlier, despite thorough cleaning of the pipework during this time, showing persistent colonisation of the sink drains by opportunistic pathogens. They also found that carbapenem resistance was highly prevalent in ICU wastewater pipes, despite low prevalence in the patient population, highlighting sink drains as long-term, persistent reservoirs of ARGs. Importantly, they were able to identify putative HGT events occurring in the hospital plumbing system. For example, the authors identified a strain of *E. cloacae* from a patient which carried two *bla*_KPC_ plasmids. The same strain of *E. cloacae* was isolated from the sink in the patient’s room 11 months later, and sequencing revealed it had gained an additional *bla*_KPC_ plasmid. This additional plasmid had also been found in a *K. pneumoniae* strain isolated from a different patient who stayed in the same room 8 months after the first patient. The authors hypothesised that the additional plasmid found in the *E. cloacae* sink drain isolate had been gained via an HGT event in the sink drain whereby the *K. pneumoniae* strain that had colonised the second patient transferred the plasmid to the strain that had originally colonised the first patient^74^. These findings are consistent with the hypothesis that bacteria colonising patients can enter hospital sinks and result in plasmid transfer—a process that is likely enhanced by the multi-species biofilm nature of the sink drain microbiome and its exposure to diverse selective pressures. Tracking such transfer events is not trivial, as observing such events requires extensive sampling, however other studies have also provided evidence of HGT in hospital sinks. Whilst tracking plasmid diversity in CPEs, a 2015 study isolated a strain of *K. pneumoniae* carrying *bla*_KPC-2_ from a patient. Four days later, two strains of *Citrobacter freundii* and *Enterobacter cloacae* were isolated from the sink in the patient’s room, both carrying the same *bla*_KPC-2_ plasmid as the patient isolate^54^. Similarly in a different study, during a hospital outbreak of *K. pneumoniae*, a strain of *Enterobacter asburiae* was isolated in a sink carrying the same *bla*_KPC-2_ plasmid as the *K. pneumoniae* outbreak strain, suggesting intergenus transfer in the hospital environment^52^. The high abundance of ARG-carrying conjugative plasmids in the hospital sink drain microbiome is implicated both by metagenomic analyses^40,51^ and by many studies that employ whole-genome sequencing on sink drain isolates^51,56,58,73–76^. The contributions of transduction and transformation to ARG exchange in the sink drain microbiome is less clear. Phage particles carrying ARGs can be highly abundant in hospital wastewater^77^, and organisms harbouring *bla*_OXA-204_ on an intact, active prophage were identified in hospital sink drain samples^78^, but the generally narrower host range of phages compared with plasmids might render the former less effective at transferring ARGs between diverse bacterial taxa. There is evidence that short pieces of exogenous extracellular DNA can be detected in sink drain biofilms for over a week^79^, and many opportunistic pathogens have some capacity for transformation^80^. As with tracking microbes, identifying MGE transfer and inferring directionality from observational sequence data is not straightforward—and the smaller size of MGEs compared with the chromosome can result in fewer nucleotide polymorphisms for tracking plasmid lineages during an outbreak^81^—but criteria have been proposed to infer dynamics of plasmid and MGE transfer using isolate sequencing data^73,82^.

Taken together, the above sections highlight (1) that the sink drain microbiome is ubiquitous and persistent, (2) that opportunistic pathogens from patients can enter and join the sink drain microbiome, (3) that ARG-encoding MGEs can transfer between pathogens and environmental organisms in greywater plumbing and that physicochemical features of this habitat can act as a stimulus for HGT, and (4) that members of the sink drain microbiome can disseminate to the patient-facing environment. Consequently, the sink drain microbiome could be considered a crucial location where clinically-relevant resistance traits are acquired by opportunistic pathogens with a high potential to cause subsequent infections.

## Interventions to reduce the risk of ARG HGT in the sink drain microbiome

Various interventions have been proposed and implemented to mitigate the risks posed by opportunistic pathogens in the sink drain microbiome, including a variety of engineering approaches such as changes to sink design, novel devices to decontaminate traps, or implementation of rigorous chemical disinfection regimes (reviewed by Kearney et al.^83^). These changes do not always have the outcomes either predicted or desired, with subsequent interventions often necessary^84^. For example, large-scale replacement of the drainage pipework to control a prolonged outbreak of carbapenem-resistant Enterobacterales resulted in significant increase in Enterobacterales and resistance gene load, as a possible response to the significant ecological disturbance represented by pipe replacement^85^. As the risks inherent with standing water cannot be fully ameliorated, the most effective protocol appears to be complete removal of sinks—and their associated drains—from patient rooms altogether^83^. However, such radical changes to hand hygiene conventions are not always viable, particularly when considering the wide healthcare context in which sink drain opportunistic pathogens and ARGs might pose a risk, such as nursing homes or the wider hospital environment.

Alternate approaches might come from a deeper understanding of the evolutionary ecology of the sink drain microbiome, and interventions based on the biotic interactions between its resident organisms^86^ (Box 1: The invasibility of the sink drain microbiome to opportunistic pathogens), and genetic elements (Box 2: Factors influencing the invasibility of a microbiome to ARG-encoding MGEs). For example, probiotics have been used as a means of controlling opportunistic pathogens in drinking water^87^, and communities recalcitrant to opportunistic pathogen invasion might ultimately be deployed to displace or impede invasion of pathogens in sink drains too. Probiotic-based surface cleaners have been evaluated in hospital settings, and one study found a significant reduction in total ARG counts in sink surface samples when using probiotic-based cleaning compared to traditional disinfectant^26^, but such approaches remain speculative in the context of influencing the function of the sink drain microbiome, and further experimental studies are necessary. Bacteriophages—viral parasites of bacteria—can be highly specific to particular hosts and thus could be a powerful tool in manipulating the sink drain microbiome to remove or reduce the risks posed by opportunistic pathogens^88,89^. An even more potent approach might stem from targeting the elements that are responsible for resistance. An unexpected diversity of phages that specifically target plasmid-harbouring bacteria have recently been catalogued, and these plasmid-dependent phages can target diverse organisms simply by virtue of their harbouring the same antibiotic resistance plasmid^90^. While bacteria can evolve resistance to phages, such mutations are commonly associated with increased susceptibility to antibiotics, or loss of the ability of the plasmid to transfer^91^, outcomes that would also ameliorate the threat of multidrug resistance gene transfer to opportunistic pathogens in the sink drain microbiome.

Box 1 The invasibility of the sink drain microbiome to opportunistic pathogens Invasibility describes how easily an introduced organism (such as an opportunistic pathogen) can establish in a target community^92^. Higher dispersal rates—i.e. increased exposure to an opportunistic pathogen—provide more opportunities for invasion, and boost invasion success by increasing invader population size^92^. There are many routes for opportunistic pathogens to disperse to sink traps (Fig. 1). To persist, an arriving invader must then find a niche. A vacant niche may be available to invaders that arrive early and possess a beneficial trait (such as a metabolic pathway) absent from the target community, but more likely the invader will be competing with one or more residents^93^. Biological features of invader and target can heighten competitive ability to influence invasion outcome, particularly the diverse arsenals of ‘interference competition’ systems that directly inhibit competitors^94^: incoming organisms expressing such systems usually make better invaders, while resident microbes with a surfeit of antagonistic interactions tend to more successfully impede invasion^95,96^. A more diverse community can be more recalcitrant to invasion, as it is more likely to contain traits that diminish any competitive advantages possessed by the invader, and more likely to express antagonistic molecules to inhibit invader growth^92^. Invasion success thus depends both on the invading organism and the target community, consistent with experimental sink drain models in which different carbapenemase-producing *Klebsiella pneumoniae* strains varied in their invasion success across biofilm communities^97^. Moreover, perturbations or disturbances to a community can favour invasions by reducing resident community population size and richness, and releasing resources that might be used by an invader^92^. Inconsistent use of sinks and irregular introduction of nutrients, biocides, pharmaceuticals, or bodily fluids^42^ could be considered perturbations that may offer potential pathogens an opportunity to become (re-)established, as has been suggested by experimental sink drain models^43,98^.

Box 2 Factors influencing the invasibility of a microbiome to ARG-encoding MGEs While most theory on microbial invasions has been developed to describe strains and species of microorganisms, analogous concepts might be applied to understand and influence the invasion of ARG-encoding MGEs in a microbiome. As with microorganisms, plasmid invasion is enhanced by dispersal: occasional disturbances to a biofilm can spread plasmid carriers across a population, increasing the chances of a plasmid accessing a new host^99^. It is conceptually straightforward to appreciate how selection for the beneficial traits harboured by MGEs, such as those encoded by ARGs, can facilitate invasion of that MGE into a novel population. Less obvious is the fact that other features of MGEs such as low fitness costs and high transmission rates can allow resistance-encoding elements to find a prolonged niche in microbiomes in the absence of regular direct selection for resistance genes^8,100,101^, a process that can be enhanced by perturbations such as nutrient availability and chemical stresses^33,99^. Similarly, it is increasingly appreciated that MGEs can have antagonistic interactions with other MGEs in a population, encoding elaborate systems to directly interfere with one another, which may impede or enhance invasion of high-impact ARG-encoding MGEs into the sink drain microbiomes^102^.

## Conclusions and perspective

Tracking and risk assessing drug resistance in the environment is not straightforward—environmental microbiomes are diverse, unobserved (and in some cases unobservable), and multitudinous. However, it is clear from genomic surveillance to date that opportunistic pathogens disseminating from the sink drain microbiome pose a threat to patients in hospital settings, and that interactions in this ‘non-infection’ habitat offer unique opportunities for such pathogens to evolve and acquire resistance traits prior to encountering chemotherapy during infection. The ability for genes to readily transfer from non-pathogenic organisms necessarily broadens our perspective on the evolution of multidrug resistance in opportunistic pathogens, highlighting the roles that MGEs can play and heightening the need to survey high-consequence MGEs, as well as pathogens per se, in the clinical environment. Finally, interventions to mitigate the threat posed by the sink drain microbiome will inevitably interact with the evolutionary ecology of pathogens, non-pathogens, and their resident MGEs in this microbiome. Studies to understand the evolutionary ecology of the sink drain microbiome—how it responds to disturbances, the principal drivers of AMR, and factors promoting gene transfer in this habitat—are necessary to improve predictions of how such interventions would play out in a real-world setting.

## Data Availability

No datasets were generated or analysed during the current study.
